# Water footprint and water productivity dynamics of Mediterranean rice under climate change and water regime scenarios

**DOI:** 10.3389/fpls.2026.1872210

**Published:** 2026-05-29

**Authors:** Alper Baydar, Yeşim Bozkurt Çolak, Burak Dalkılıç, Yusuf Çelik

**Affiliations:** 1 Department of Biosystems Engineering, Faculty of Agriculture, Siirt University, Siirt, Türkiye; 2 Department of Biosystems Engineering, Faculty of Agriculture, Malatya Turgut Özal University, Malatya, Türkiye; 3 Department of Plant and Animal Production, Silifke Vocational School, Mersin University, Silifke, Türkiye

**Keywords:** blue water footprint, climate change, DSSAT, green water footprint, water productivity

## Abstract

**Introduction:**

Rice production is highly dependent on water availability and is increasingly exposed to climate-driven changes in evapotranspiration, precipitation patterns, and irrigation demand.

**Methods:**

This study evaluated future rice water footprint and water productivity responses using DSSAT-CERES-Rice outputs previously calibrated and validated for Mediterranean conditions in Türkiye. The validation-year full-irrigation reference was used to represent future irrigated conditions in the present water footprint assessment. Simulations were evaluated using three GCMs, HadGEM2-ES, GFDL-ESM2M, and MPI-ESM-MR, two emission scenarios, RCP 4.5 and RCP 8.5, and three projection periods: 2016–2040, 2041–2070, and 2071–2098.

**Results:**

For irrigated conditions, mean green + blue water footprint was highest in 2016–2040, reaching 1250 m³ t⁻¹ for RCP 4.5 and 1056 m³ t⁻¹ for RCP 8.5, but remained lower in the late-future period, reaching 674 and 668 m³ t⁻¹, respectively, by 2071–2098. For rainfed conditions, mean green water footprint was considerably higher in the near-future period, reaching 6401 m³ t⁻¹ for RCP 4.5 and 4610 m³ t⁻¹ for RCP 8.5, mainly because limited growing-season rainfall and increased water stress reduced simulated yields under non-irrigated conditions. These values remained below the near-future levels in the late-future period, reaching 1869 and 2608 m³ t⁻¹, respectively, in 2071–2098. Irrigation water productivity ranged from 0.88 to 9.37 kg m⁻³, indicating strong sensitivity to irrigation water use and yield response, particularly in scenarios with low simulated irrigation requirements.

**Discussion:**

Overall, future rice water footprint was controlled not only by evapotranspiration, but also by yield response, irrigation regime, and GCM-specific climate responses. The study demonstrates that validated DSSAT outputs can be used not only for future yield projection, but also for water footprint assessment, providing a useful framework for future rice water management under climate change.

## 1 Introduction

Rice production is unique in terms of water use because rice productivity is influenced not only by seasonally available water, but also by how consistently this water is available throughout the growing cycle. The importance of water for rice is expected to become even more critical in Mediterranean climates, where rice is commonly cultivated under high evaporative demand and limited precipitation during the growing season. Future changes in temperature, precipitation distribution, and atmospheric evaporative demand may affect rice production through two interrelated pathways: the quantity of water used by the crop and the yield generated from that water. Therefore, assessing future rice production only through yield projections may not fully represent its sustainability under climate change.

Water footprint assessment enables crop water consumption to be evaluated in relation to crop production rather than only as total water use. In the widely used water footprint framework, green water refers to precipitation-derived water consumed by crops, blue water refers to consumptive irrigation water use from surface or groundwater resources, and grey water refers to the volume of freshwater required to dilute pollutants to acceptable levels (Mekonnen and Hoekstra, 2011). Distinguishing between green and blue components is particularly important in irrigated rice systems because precipitation may still contribute to crop evapotranspiration even when irrigation is applied. Therefore, evaluating green and blue water components separately provides a more comprehensive assessment of crop water use than considering total water consumption alone.

One of the major issues in crop water footprint studies is that evapotranspiration and water footprint do not always change in the same direction. Evapotranspiration represents crop water consumption; however, water footprint expresses this consumption per unit of yield. Therefore, a scenario with greater evapotranspiration may still result in a lower water footprint if yield increases sufficiently. Conversely, low-yielding scenarios may produce high water footprints even when seasonal evapotranspiration is not particularly high. This point is important for future climate assessments because temperature increase, CO_2_ concentration, precipitation variability, and irrigation response may affect both crop growth and water balance processes simultaneously. Therefore, yield, evapotranspiration, and water source components should be evaluated together when interpreting future responses of rice water footprint (Mialyk et al., 2024).

Process-based crop models are useful for examining these changes because they can integrate climate data, soil characteristics, cultivar information, crop developmental processes, and growing-season management practices within a single simulation framework. DSSAT has been widely used to simulate crop development and soil–plant–atmosphere interactions under different environmental conditions and management practices (Jones et al., 2003). For rice, DSSAT-CERES-Rice can generate the main outputs required for a model-based water footprint assessment, particularly simulated yield and evapotranspiration. When generated using a calibrated and validated model, these outputs can be converted into water productivity and water footprint indicators. This conversion of model outputs into water-related indicators is particularly important under future climate scenarios, where direct field observations are not available and crop responses depend on the interaction between plant development and water balance processes (Shrestha et al., 2017).

Previous rice-focused studies indicate that climate change may affect rice water footprint differently depending on region, climate scenario, production condition, and model structure. Water footprint responses have been evaluated for rice production in different climatic and hydrological contexts, including Thailand, China, Italy, and arid environments, showing that future changes in yield and evapotranspiration can alter the amount of water consumed per unit of production (Bocchiola, 2015; Yang et al., 2018; Arunrat et al., 2020; Zheng et al., 2020; Elsadek et al., 2025).

Recent studies also demonstrate that water footprint analysis provides additional value when linked with water productivity and water management indicators. Different irrigation methods can alter the relative contribution of green and blue water components. Basin-scale assessments also indicate that climate-related changes in precipitation and crop productivity may affect both yield and water footprint. In rice-based production systems, water footprint indicators have been used to evaluate environmental performance beyond yield alone (Zhou et al., 2022; Yue et al., 2022; Lin and Chiang, 2025).

Although progress has been made in modelling rice water footprint trends under different climatic and management conditions, future water footprint responses of Mediterranean rice remain insufficiently evaluated using validated crop model outputs. This is particularly important for Mediterranean rice production because high evaporative demand during the growing season, limited seasonal water availability, and uncertainty in future climate conditions may jointly affect evapotranspiration, yield, and the balance between green and blue water components. Therefore, a combined assessment of yield, evapotranspiration, green water footprint, blue water footprint, and water productivity can provide a more comprehensive understanding of future rice water use under climate change.

The objective of this study was to evaluate future water footprint dynamics of Mediterranean rice production under climate change using DSSAT-CERES-Rice-derived scenario outputs. Specifically, the study aimed to: (i) quantify green and blue water footprint components under irrigated conditions, (ii) assess green water footprint under rainfed conditions, (iii) compare future irrigated green + blue water footprint with the full-irrigation reference condition, and (iv) examine yield–evapotranspiration–water productivity relationships across GCMs, RCP scenarios, and projection periods. HadGEM2-ES, GFDL-ESM2M, and MPI-ESM-MR were used as TSMS-provided climate projections dynamically downscaled with RegCM4.3.4 at 20 km spatial resolution and bias-corrected for Türkiye under RCP 4.5 and RCP 8.5 scenarios, providing a consistent multi-model dataset for the Mediterranean scenario analysis. Accordingly, this study focuses on the consumptive green and blue water footprint components estimated from DSSAT-CERES-Rice yield and evapotranspiration outputs, while the grey water footprint was considered outside the scope of the present analysis.

## 2 Materials and methods

### 2.1 Study basis, experimental site and climate scenario framework

DSSAT-CERES-Rice yield/model-output information from Baydar (2026) was used as input for new water footprint and water productivity calculations in the present study. The original field experiment was conducted during the 2019–2020 growing seasons at the Tarsus location of Alata Horticultural Research Institute in Mersin, Türkiye. The experimental site is located in the Mediterranean region of Türkiye and represents a semi-arid agricultural environment where crop production is exposed to high seasonal evaporative demand and variable water availability during the growing period. The area is characterized by hot and dry summers, with precipitation mainly concentrated outside the summer growing season.

In the modelling framework, DSSAT-CERES-Rice was calibrated using phenological observations collected in 2019 and validated using independent field data from 2020. The field experiment included three irrigation coefficients, and cultivar genetic coefficients were estimated using the full-irrigation treatment. Model validation was performed using yield, biomass, and leaf area index. The reported validation statistics showed strong model performance for yield simulation, with nRMSE values below 10% and Dindex values of 0.99 across irrigation levels.

In this study, the validated DSSAT-CERES-Rice outputs were used as the basis for future water footprint and water productivity assessment. The full-irrigation reference from the validation framework was used to represent future irrigated conditions in this study. This approach ensured methodological continuity between the crop simulation framework and the present water footprint analysis.

Future simulations were evaluated using three global climate models: HadGEM2-ES, GFDL-ESM2M, and MPI-ESM-MR. Two emission scenarios, RCP 4.5 and RCP 8.5, were considered across three projection periods: 2016–2040, 2041–2070, and 2071–2098. The soil profile used in the DSSAT-CERES-Rice simulations was defined from the measured soil characteristics of the experimental site and was assumed to remain unchanged across the future projection periods. Long-term changes in soil organic matter, soil degradation, and salinity accumulation were not dynamically modelled in the present framework. The climate datasets were dynamically downscaled using RegCM4.3.4 at 20 km spatial resolution by the Turkish State Meteorological Service (TSMS). Bias correction was performed using the additive delta-change method based on the 1970–2000 reference climate series and the observed daily meteorological data for the 2019–2020 experimental years. Atmospheric CO_2_ concentrations varied among future simulations according to the RCP-specific concentration pathways. For each GCM–RCP–period combination, the corresponding CO_2_ concentration was entered into DSSAT-CERES-Rice as an input parameter. The representative CO_2_ concentrations used in the underlying DSSAT simulations were approximately 436, 509, and 534 ppm under RCP 4.5 and 450, 593, and 846 ppm under RCP 8.5 for 2016–2040, 2041–2070, and 2071–2098, respectively. Thus, simulated yield, evapotranspiration, and water-balance outputs were generated under scenario-specific atmospheric CO_2_ conditions within the DSSAT-CERES-Rice framework. The same climate scenario structure was used in the present study to derive future green water footprint, blue water footprint, green + blue water footprint, irrigation water productivity, and evapotranspiration-based water productivity indicators.

### 2.2 Estimation of green and blue water footprint components

Water footprint indicators were calculated to express simulated water consumption per unit of rice yield. The calculation focused on the consumptive green and blue components of the water balance, which represent precipitation-derived and irrigation-derived water use, respectively. Accordingly, the reported indicators describe the green and blue dimensions of future rice water use.

Under irrigated conditions, green water footprint (WFG), blue water footprint (WFB), and green + blue water footprint (WFG+B) were calculated. Under rainfed conditions, no irrigation water was applied; therefore, WFG was considered as the relevant consumptive water footprint component. The component-based calculation followed the crop water footprint approach, in which green and blue crop water use components are related to yield (Chapagain and Hoekstra, 2011; Mekonnen and Hoekstra, 2011).

For irrigated conditions, the green water component was derived from the rainfed evapotranspiration value corresponding to the same GCM, RCP scenario, and projection period, while the blue water component was calculated as the difference between irrigated and rainfed evapotranspiration. The following equations were used:


WFG=(10×ET_green)/Y

WFB=(10×ET_blue)/Y

WFG+B=WFG+WFB

where WFG is green water footprint (m³ t^-^¹), WFB is blue water footprint (m³ t^-^¹), ETgreen and ETblue are expressed in mm, and Y is yield (t ha^-^¹). The coefficient 10 converts water depth from mm over one hectare into m³ ha^-^¹. The present methodological framework was confined to consumptive green and blue water footprint components derived from DSSAT-CERES-Rice yield and evapotranspiration outputs, since grey water footprint estimation requires pollutant-related and water-quality variables beyond the scope of the DSSAT-based dataset used in this study.

### 2.3 Calculation of water productivity indicators

To assess future rice water use in terms of both consumption and productivity, water productivity indicators were calculated alongside water footprint indicators. While water footprint expresses water consumption per unit of yield, water productivity expresses yield obtained per unit of water input or water consumption. Therefore, these indicators were evaluated together to interpret the relationship between water use and rice production under future climate scenarios.

Irrigation water productivity (IWP) was calculated only for irrigated conditions. IWP was expressed as yield per unit of irrigation water use, following the definition of irrigation water productivity as the yield produced per unit of irrigation water use (Li et al., 2016). IWP was calculated as:


$$IWP=\frac{Y\times 100}{I}$$

where IWP is irrigation water productivity (kg m^-^³), Y is yield (t ha^-^¹), and I is seasonal irrigation amount (mm). The coefficient 100 converts yield in t ha^-^¹ and irrigation depth in mm into kg m^-^³.

Evapotranspiration-based water productivity (WPET) was calculated for both irrigated and rainfed conditions. This indicator expresses yield per unit of seasonal actual evapotranspiration and follows the WPET calculation approach in which yield is divided by ETa (Zeleke and Nendel, 2024). WPET was calculated as:


$$WP_{ET}=\frac{Y\times 100}{ETa}$$

where WPET is evapotranspiration-based water productivity (kg m^-^³), Y is yield (t ha^-^¹), and ETa is seasonal actual evapotranspiration (mm). The coefficient 100 converts yield in t ha^-^¹ and evapotranspiration depth in mm into kg m^-^³.

### 2.4 Comparative analysis of water footprint and productivity indicators

The calculated indicators were organized according to production condition, GCM, RCP scenario, and projection period. For each GCM–RCP–period combination, yield, evapotranspiration, irrigation amount, water footprint components, and water productivity indicators were evaluated using the equations described above. Irrigated conditions were assessed through WFG, WFB, WFG+B, IWP, and WPET, whereas rainfed conditions were assessed through WFG and WPET.

GCM-specific values were retained to show model-dependent variation among HadGEM2-ES, GFDL-ESM2M, and MPI-ESM-MR. In addition, three-GCM mean values and minimum–maximum ranges were calculated for each RCP–period combination. This structure allowed the interpretation of both average future tendencies and inter-model variability in water footprint and productivity indicators.

The comparative analysis focused on four main aspects: changes in green + blue water footprint under irrigated conditions, green water footprint response under rainfed conditions, the relative contribution of green and blue components to WFG+B, and the relationship between yield, evapotranspiration, IWP, and WPET. This arrangement enabled future rice water use to be evaluated through both footprint-based and productivity-based indicators within the same climate scenario framework.

In addition to descriptive comparisons, a non-parametric statistical analysis was performed using GCM-specific values as paired model outputs. Differences among projection periods were evaluated separately for each RCP scenario using the Friedman test (Friedman, 1937), whereas differences between RCP 4.5 and RCP 8.5 within each projection period were evaluated using the paired Wilcoxon signed-rank test (Wilcoxon, 1945).

## 3 Results

### 3.1 Future green + blue water footprint under irrigated conditions

Yield, evapotranspiration, irrigation amount, irrigation water productivity, and green–blue water footprint indicators for irrigated rice conditions are presented in **Table 1**. The green + blue water footprint (WFG+B) differed among GCMs, RCP scenarios, and projection periods. In 2016–2040, WFG+B for RCP 4.5 ranged from 1181.88 m³ t^-^¹ in MPI-ESM-MR to 1323.86 m³ t^-^¹ in GFDL-ESM2M, whereas the RCP 8.5 scenario produced values between 673.05 m³ t^-^¹ in HadGEM2-ES and 1280.07 m³ t^-^¹ in GFDL-ESM2M. Arunrat et al. (2020) reported scenario-dependent rice total water footprint responses under future RCP conditions, with projected total WF changes ranging from −10.0 to −43.0% for individual farming and from −0.5 to −67.0% for large-scale farming under RCP 4.5, and from −26.5 to 63.3% and −51.1 to 60.0%, respectively, under RCP 8.5.

**Table 1:** Irrigated rice yield, water use and green–blue water footprint indicators under RCP 4.5 and RCP 8.5.

Periods	GCM	Irrigated conditions													
		RCP 4.5							RCP 8.5						
		Yield (t ha^-^¹)	ET (mm)	Irrigation (mm)	IWP (kg m^-^³)	WFG (m³ t^-^¹)	WFB (m³ t^-^¹)	WFG+B (m³ t^-^¹)	Yield (t ha^-^¹)	ET (mm)	Irrigation (mm)	IWP (kg m^-^³)	WFG (m³ t^-^¹)	WFB (m³ t^-^¹)	WFG+B (m³ t^-^¹)
2016–2040	HadGEM2-ES	5.37	667.6	111.0	4.84	817.06	426.60	1243.67	5.27	354.9	56.3	9.37	653.33	19.72	673.05
	GFDL-ESM2M	5.13	679.8	455.0	1.13	690.56	633.30	1323.86	5.31	680.1	450.0	1.18	680.22	599.85	1280.07
	MPI-ESM-MR	6.09	720.0	630.0	0.97	360.97	820.91	1181.88	6.00	728.4	678.8	0.88	373.00	841.00	1214.00
2041–2070	HadGEM2-ES	16.93	859.4	383.3	4.42	501.92	5.85	507.77	8.08	697.5	440.0	1.84	515.10	347.93	863.03
	GFDL-ESM2M	9.56	804.6	575.8	1.66	461.24	380.48	841.72	9.56	761.8	529.0	1.81	452.35	344.60	796.95
	MPI-ESM-MR	12.31	804.5	731.3	1.68	199.24	454.46	653.69	12.80	817.3	753.3	1.70	192.35	445.92	638.27
2071–2098	HadGEM2-ES	16.50	965.7	576.0	2.86	585.45	0.00	585.45	12.88	832.1	640.0	2.01	372.40	273.44	645.84
	GFDL-ESM2M	13.43	1114.8	874.0	1.54	404.80	425.35	830.14	13.46	1014.9	762.0	1.77	424.26	329.69	753.96
	MPI-ESM-MR	14.03	852.5	764.0	1.84	215.17	392.42	607.58	15.06	910.2	868.0	1.74	198.01	406.37	604.38

ET, seasonal evapotranspiration; IWP, irrigation water productivity. WFG and WFB refer to green and blue water footprint, respectively, while WFG+B denotes the sum of green and blue water footprint components. For irrigated conditions, green and blue water footprints were calculated based on the partitioning of crop evapotranspiration into green and blue water components.

**Figure 1:**
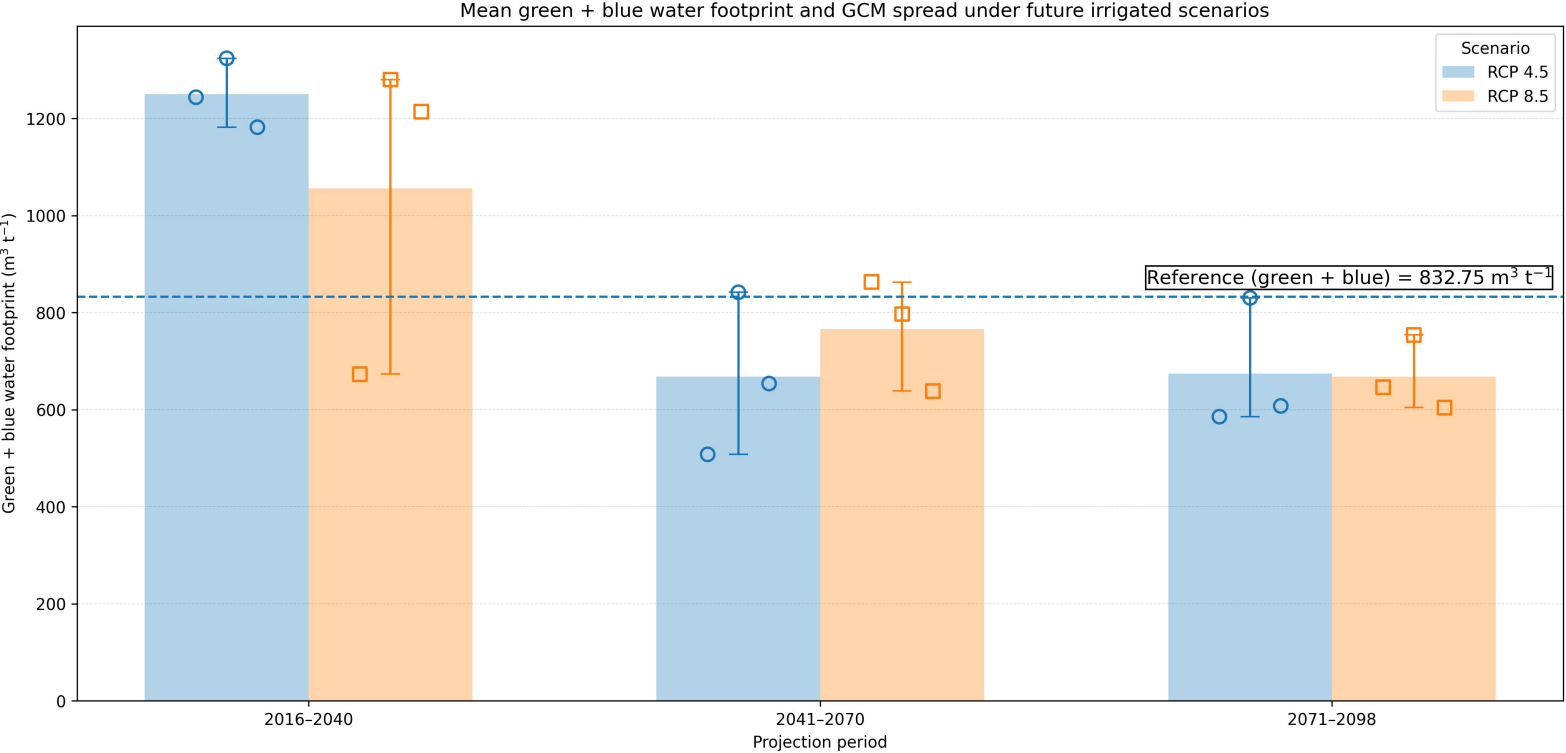
Mean green + blue water footprint and GCM spread under future irrigated scenarios. Bars show the three-GCM mean, whiskers indicate the minimum–maximum range among GCMs, and symbols represent individual GCM outputs. The dashed line indicates the validation-year full-irrigation reference.

In 2041–2070, WFG+B values were generally lower than those recorded in the near-future period. For RCP 4.5, the lowest value was calculated for HadGEM2-ES at 507.77 m³ t^-^¹, while the highest value was obtained for GFDL-ESM2M at 841.72 m³ t^-^¹. In the same period, RCP 8.5 showed a minimum WFG+B of 638.27 m³ t^-^¹ for MPI-ESM-MR and a maximum of 863.03 m³ t^-^¹ for HadGEM2-ES. In 2071–2098, WFG+B remained below the near-future level across all model–scenario combinations. For RCP 4.5, the minimum and maximum values were 585.45 m³ t^-^¹ for HadGEM2-ES and 830.14 m³ t^-^¹ for GFDL-ESM2M, respectively. For RCP 8.5, WFG+B varied from 604.38 m³ t^-^¹ for MPI-ESM-MR to 753.96 m³ t^-^¹ for GFDL-ESM2M. The mean green + blue water footprint values and GCM ranges are shown in **Figure 1**. The highest mean WFG+B was observed in 2016–2040, followed by lower values in the mid- and late-future periods. Mean WFG+B values reduced from 1250 to 674 m³ t^-^¹ under RCP 4.5 and from 1056 to 668 m³ t^-^¹ for RCP 8.5 between the near- and late-future periods. Deihimfard et al. (2022) reported that future climate reduced total water footprint by 8% in irrigated wheat and 11% in rainfed wheat, mainly through increased yield and reduced water consumption. These findings support the view that lower water footprint values may result from combined changes in crop water consumption and yield response rather than from evapotranspiration alone.

### 3.2 Green and blue component contributions under irrigated conditions

**Figure 2:**
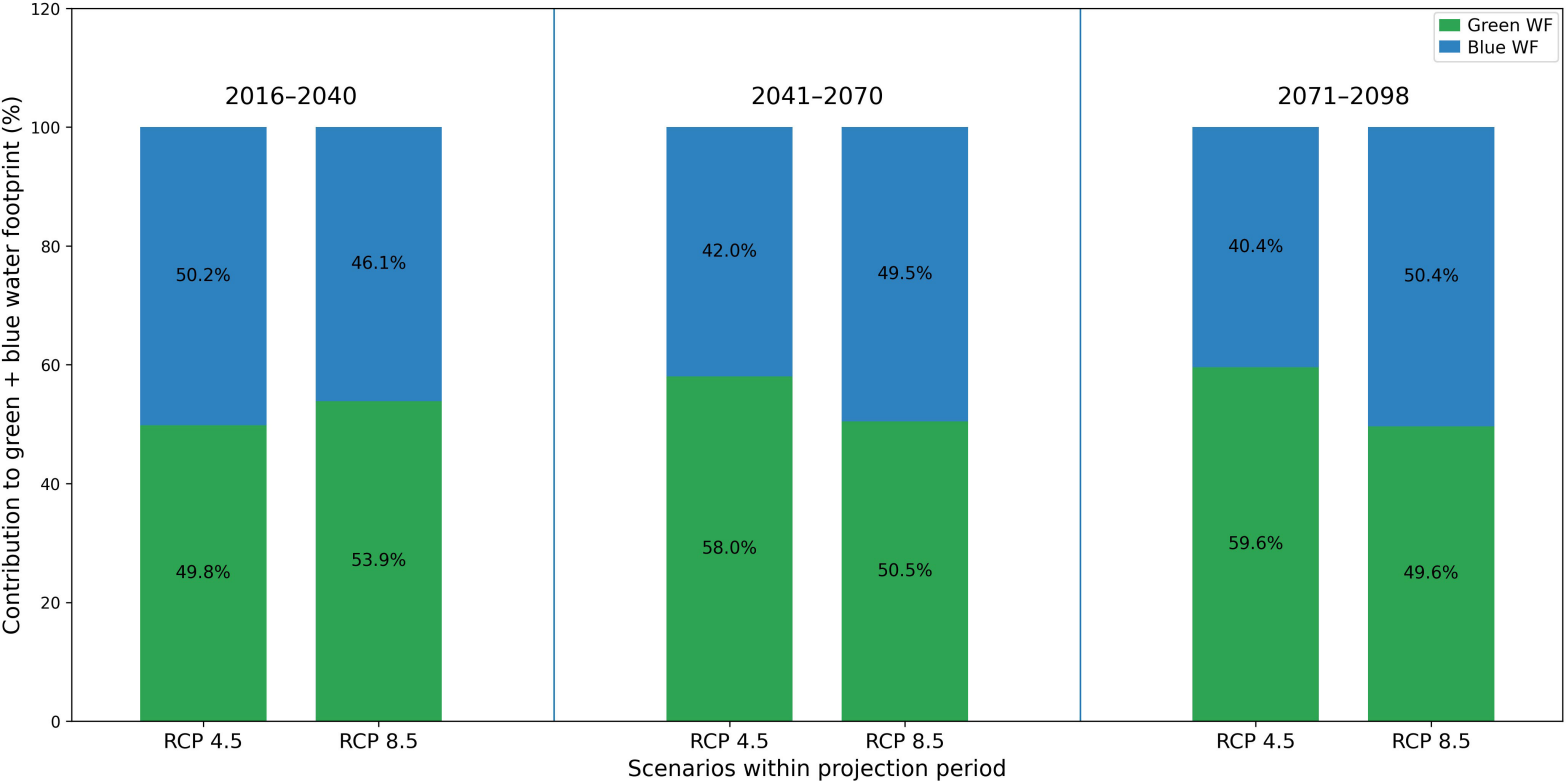
Relative contributions of green and blue components to the green + blue water footprint under future irrigated scenarios. Values inside the bars indicate percentage contributions.

The relative contributions of green and blue components to WFG+B under irrigated conditions are shown in **Figure 2**. In 2016–2040, the RCP 4.5 scenario showed an almost balanced green–blue distribution, with shares of 49.8% and 50.2% in WFG+B, respectively. For RCP 8.5, the green share was slightly higher than the blue share, at 53.9% and 46.1%, respectively. This pattern indicates that both precipitation-derived and irrigation-derived water remained important components of the consumptive water footprint under future irrigated conditions. Although the grey water component was not included in the present analysis, the presence of substantial green and blue components is consistent with the rice water footprint structure reported by Chapagain and Hoekstra (2011). In that study, the global rice water footprint consisted of 48% green, 44% blue, and 8% grey water footprint.

In 2041–2070, the share of the green water component increased to 58.0% under RCP 4.5, whereas RCP 8.5 showed nearly equal green and blue contributions, with 50.5% green and 49.5% blue. In 2071–2098, RCP 4.5 was more clearly dominated by the green component, which accounted for 59.6% of WFG+B, while RCP 8.5 showed a slight dominance of the blue component, with 50.4%. The shift in green–blue contribution across periods is also consistent with the observation of Chapagain and Hoekstra (2011) that the ratio of green to blue water in rice production varies strongly over time and space. Overall, these results indicate that the water footprint of irrigated rice should not be interpreted only through irrigation water use, because the green component remained an important part of WFG+B across future scenarios.

### 3.3 Green water footprint under rainfed conditions

Yield, evapotranspiration, and green water footprint values for rainfed rice conditions are presented in **Table 2**. Rainfed rice showed considerably higher WFG values than irrigated WFG+B in the near-future period. During 2016–2040, RCP 4.5 WFG varied between 3947.94 m³ t^-^¹ in MPI-ESM-MR and 8040.82 m³ t^-^¹ in GFDL-ESM2M. In the RCP 8.5 scenario, the minimum and maximum WFG values were 2434.63 m³ t^-^¹ in HadGEM2-ES and 5952.13 m³ t^-^¹ in MPI-ESM-MR, respectively. These high values were mainly associated with low simulated yields under rainfed conditions. The DSSAT WTH input for the MPI-ESM-MR RCP 8.5 near-future rainfed simulation showed a 41-day rainless period during the growing season, with a mean Tmax of 38.9 °C and a maximum Tmax of 49.3 °C. This dry–hot episode overlapped with the model-simulated anthesis period around day 248 of the year, indicating that water and heat stress during a sensitive reproductive stage contributed to the very low simulated rainfed yield. Behera et al. (2023) reported that the total water footprint of rainfed rice varieties increased by 71.8–101.9% under RCP 4.5 and by 66.6–67.3% for RCP 8.5 compared with their baseline period.

**Table 2:** Period-based rice yield, evapotranspiration and green water footprint under rainfed conditions.

Periods	GCM	Rainfed conditions					
		RCP 4.5			RCP 8.5		
		Yield (t ha^-^¹)	ET (mm)	WFG (m³ t^-^¹)	Yield (t ha^-^¹)	ET (mm)	WFG (m³ t^-^¹)
2016–2040	HadGEM2-ES	0.61	438.6	7213.82	1.42	344.5	2434.63
	GFDL-ESM2M	0.44	354.6	8040.82	0.66	361.4	5442.77
	MPI-ESM-MR	0.56	219.9	3947.94	0.38	223.8	5952.13
2041–2070	HadGEM2-ES	3.41	849.5	2490.47	4.86	416.3	857.11
	GFDL-ESM2M	1.50	440.9	2929.57	1.69	432.4	2561.61
	MPI-ESM-MR	1.28	245.2	1908.17	1.23	246.3	2002.44
2071–2098	HadGEM2-ES	4.39	965.7	2197.27	5.14	479.8	934.19
	GFDL-ESM2M	3.38	543.6	1610.67	1.61	571.1	3547.20
	MPI-ESM-MR	1.68	301.9	1798.09	0.89	298.2	3343.05

ET, seasonal evapotranspiration; WFG, green water footprint. Under rainfed conditions, no irrigation water was applied; therefore, the blue water footprint component was not reported. The reported water footprint represents only the green water footprint component.

Rainfed WFG was lower in 2041–2070 than in the near-future period. In the RCP 4.5 simulations, the lowest value was calculated for MPI-ESM-MR, at 1908.17 m³ t^-^¹, while the highest value was obtained for GFDL-ESM2M, at 2929.57 m³ t^-^¹. The RCP 8.5 scenario presented a lower minimum value in HadGEM2-ES, at 857.11 m³ t^-^¹, and a maximum value of 2561.61 m³ t^-^¹ in GFDL-ESM2M. By 2071–2098, mean WFG values were 1869 m³ t^-^¹ in RCP 4.5 and 2608 m³ t^-^¹ in RCP 8.5. The difference between the near-future and later periods remained more pronounced under rainfed conditions than under irrigated conditions, indicating the strong influence of yield response on WFG when irrigation water was not applied. Yue et al. (2022) also showed that water footprint variations were closely associated with changes in crop yield and crop water consumption.

**Figure 3:**
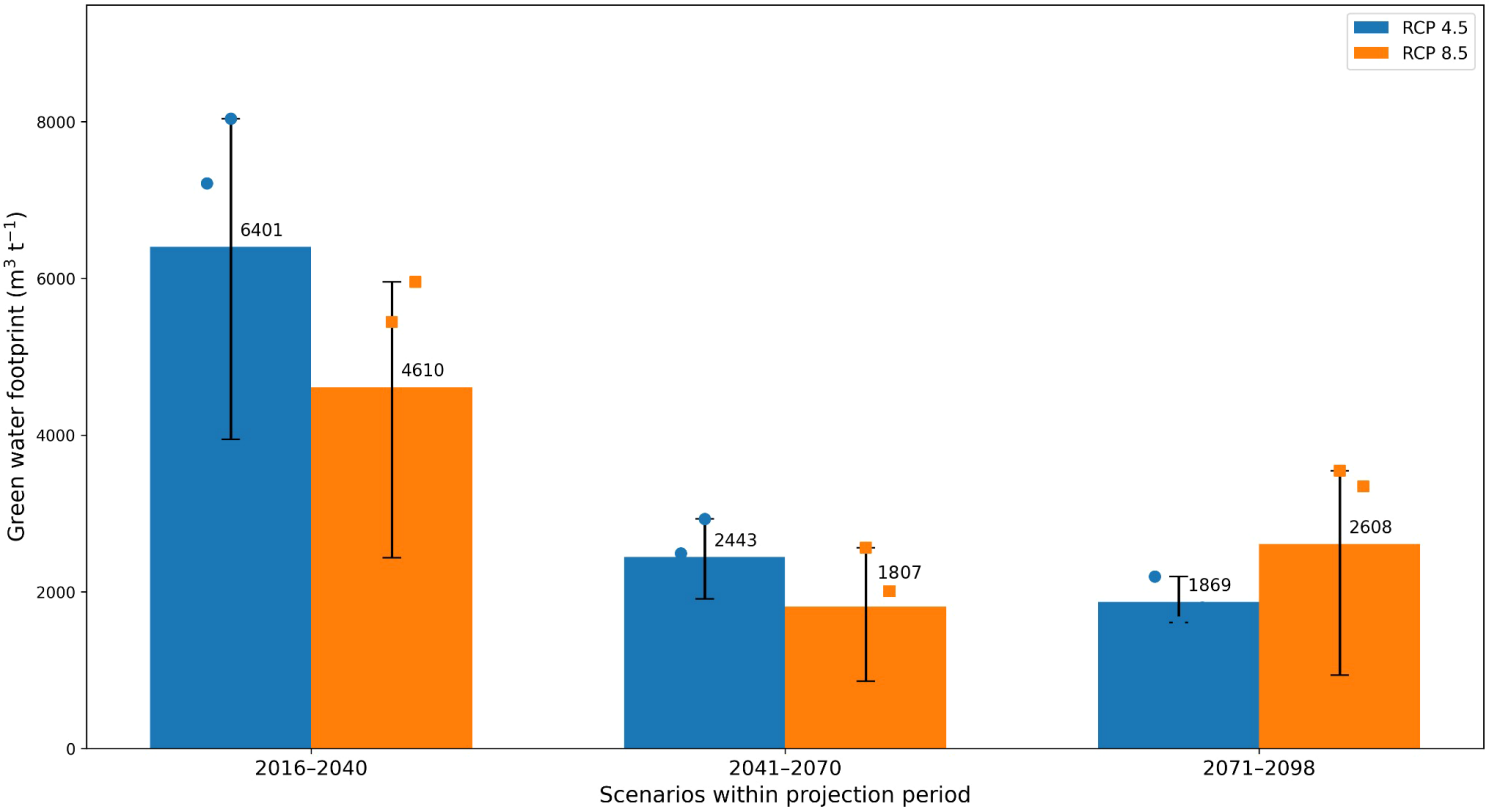
Mean green water footprint and GCM spread under future rainfed scenarios. Bars show the three-GCM mean, whiskers indicate the minimum–maximum range among GCMs, and symbols represent individual GCM outputs.

The mean green water footprint values and GCM ranges under rainfed conditions are shown in **Figure 3**. The graphical summary highlights a marked decline in mean WFG from the near-future to the mid-future period under both RCP scenarios. Under RCP 4.5, this decline continued into the late-future period, whereas under RCP 8.5 the mean WFG increased again in 2071–2098 relative to 2041–2070. The GCM range was widest in 2016–2040, particularly under RCP 4.5, showing that rainfed green water footprint was highly sensitive to model-specific yield and evapotranspiration responses in the near-future period. Overall, this pattern supports the interpretation that the high rainfed WFG values in the near-future period were primarily driven by low simulated productivity rather than by evapotranspiration alone.

### 3.4 Yield–evapotranspiration–water footprint relationships

**Figure 4:**
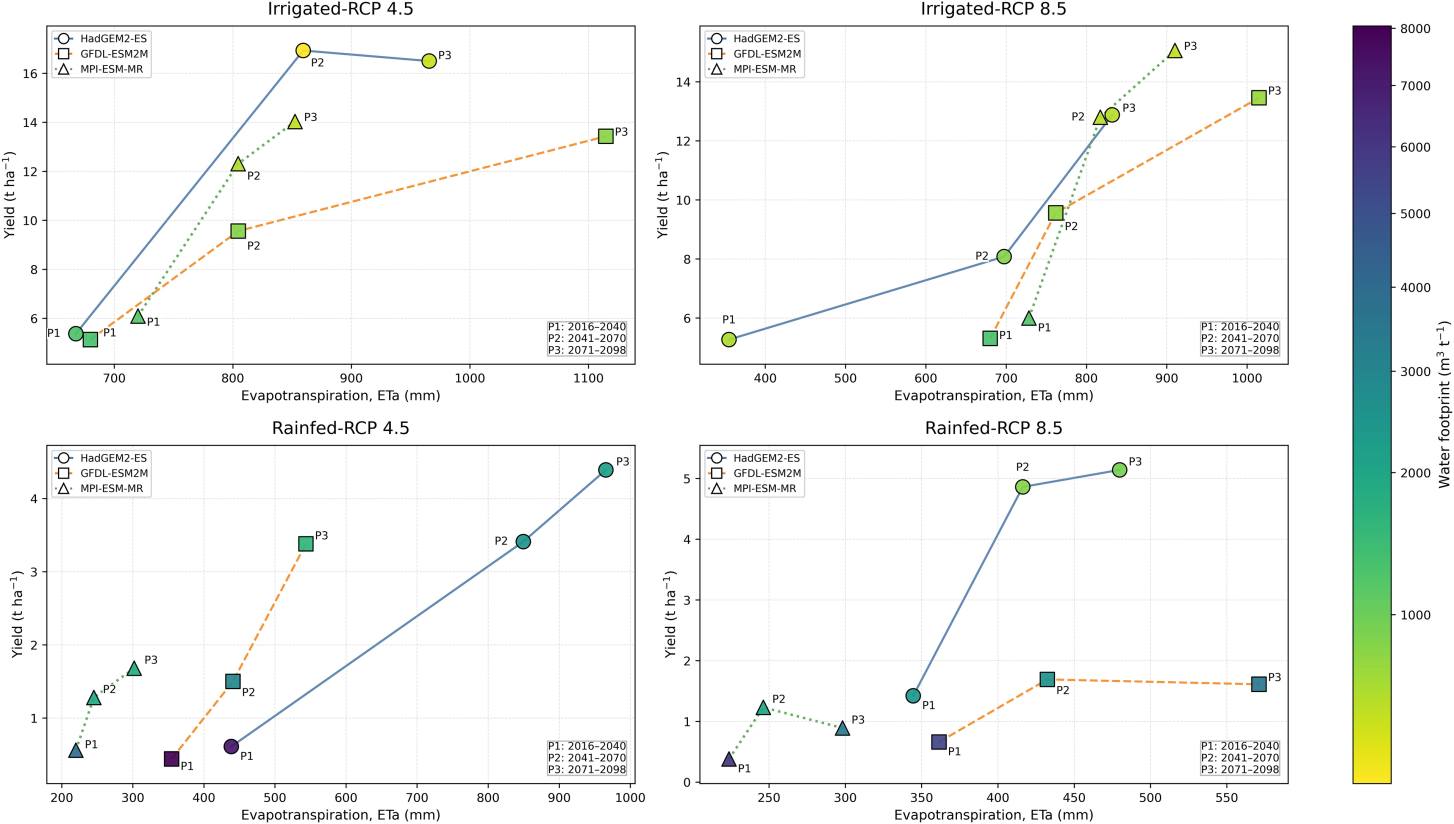
Future yield–evapotranspiration patterns under irrigated and rainfed conditions. Marker shapes denote GCMs, P1–P3 indicate projection periods, and the color scale represents the corresponding water footprint value.

The relationships among yield, evapotranspiration, and water footprint across production conditions, RCP scenarios, GCMs, and projection periods are illustrated in **Figure 4**. Under irrigated conditions, higher ETa was often associated with higher yield, particularly in some mid- and late-future simulations. However, water footprint values were not determined by ETa alone. Some simulations with relatively high ETa produced lower WFG+B values because yield increased more strongly than crop water consumption. Under rainfed conditions, the yield–ETa relationship was more variable. In 2016–2040, low yield combined with moderate ETa resulted in high WFG values. In later periods, improved simulated yield in several GCM–scenario combinations reduced WFG despite relatively high ETa, although this response was not uniform across all simulations. Mean WFG+B in irrigated rice decreased from 2016–2040 to 2071–2098 by 46.1% in the RCP 4.5 scenario and by 36.7% in RCP 8.5. In rainfed rice, the reduction in mean WFG was more pronounced over the same period, at 70.8% for RCP 4.5 and 43.4% for RCP 8.5. These changes showed that water footprint responses were shaped by yield–water interactions rather than evapotranspiration alone. Using CERES-Rice, Shrestha et al. (2017) also reported cultivar-dependent rice water footprint responses across RCP 4.5 and RCP 8.5 scenarios. In their study, the water footprints of KDML-105 and RD-6 increased by 56.5–92.2% and 27.5–29.7% in the RCP 4.5 simulations, respectively, while the RCP 8.5 simulations resulted in increases of 71.4–76.5% and 27.9–37.6%. By contrast, ChaiNat-1 showed reductions of 39.4–42.1% and 31.7–38.5%, respectively. Elsadek et al. (2025) also evaluated rice yield, evapotranspiration, and total water footprint together using SSP-based climate pathways. They reported that total water footprint increased by 2.88–17.13% when the CO_2_ effect was excluded, but decreased by 15.45–34.50% when this effect was included.

### 3.5 Water productivity indicators

**Figure 5:**
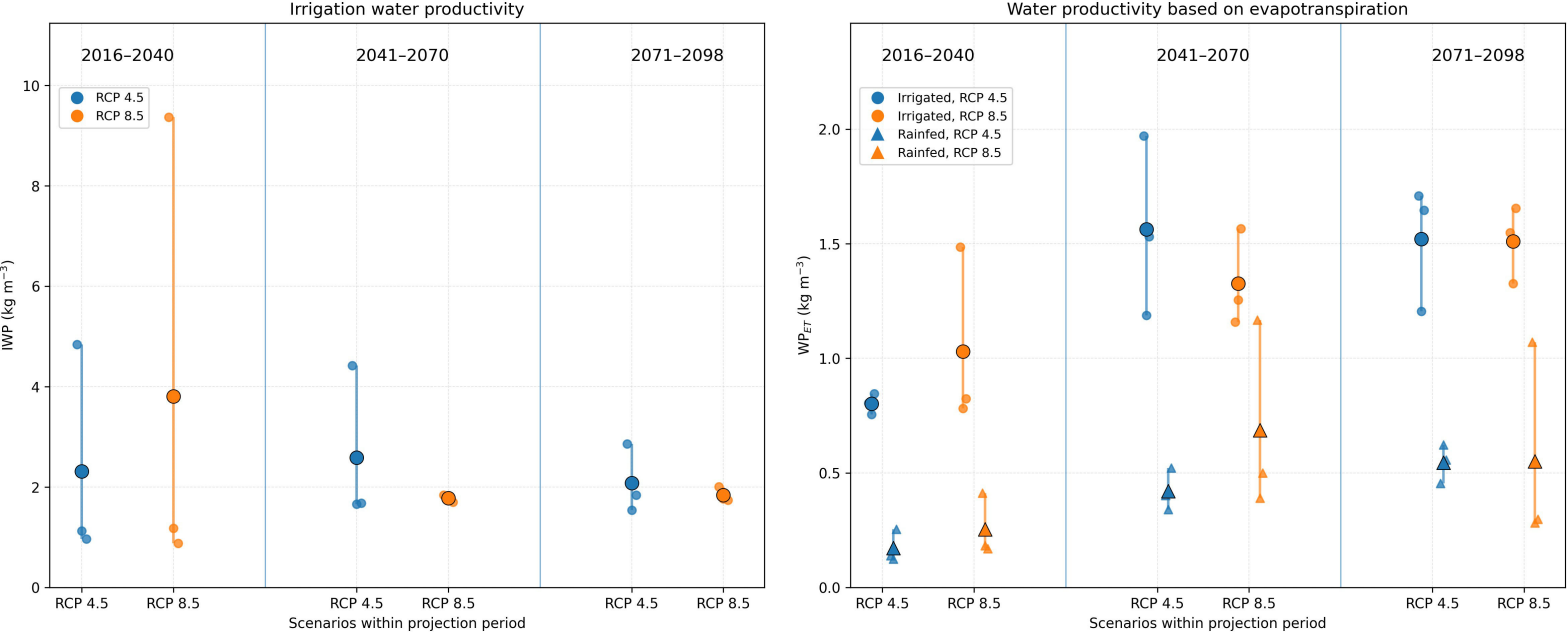
Future water productivity indicators under irrigated and rainfed conditions: irrigation water productivity and water productivity based on evapotranspiration. Large symbols show three-GCM means, small symbols indicate individual GCM outputs, and whiskers represent the minimum–maximum range among GCMs.

Irrigation water productivity and evapotranspiration-based water productivity responses are shown in **Figure 5**. In interpreting these indicators, IWP refers to yield per unit of seasonal irrigation water applied, whereas WPET represents yield per unit of seasonal evapotranspiration. IWP varied widely under irrigated conditions. The maximum IWP value was mainly associated with the low simulated irrigation requirement under the HadGEM2-ES RCP 8.5 near-future scenario. The corresponding bias-corrected DSSAT WTH climate input showed high growing-season rainfall, while the simulated irrigation requirement was also affected by daily water-balance conditions, crop water demand, evapotranspiration, and rainfall distribution. This unusually high IWP value should not be interpreted as evidence that irrigation demand would generally become negligible under Mediterranean warming. Rather, it reflects a HadGEM2-ES-specific wet signal in the bias-corrected climate input, consistent with Türkiye-scale RegCM4 projections reporting increased summer precipitation under HadGEM2-ES RCP 8.5 during 2016–2040, except for the western Mediterranean region, while MPI-ESM-MR and GFDL-ESM2M generally projected decreasing summer precipitation patterns (Demircan et al., 2017). Monaco and Sali (2018) reported that rice IWP varied from 0.09 to 8.10 kg m^-^³ across experimental data from 51 studies, with mean and median values of 1.36 and 0.85 kg m^-^³, respectively. The wide IWP range in the current simulations was therefore associated with both irrigation amount and yield response. Because IWP is calculated from applied irrigation water, it should be evaluated together with evapotranspiration-based indicators rather than as a stand-alone measure.

WPET provided a different perspective on water productivity because it relates yield to evapotranspiration rather than to irrigation amount. Under irrigated conditions, WPET was generally higher in the mid- and late-future periods than in the near-future period, particularly where yield response improved. Under rainfed conditions, WPET was lowest in the near-future period due to low simulated yields and increased in later periods when yield performance improved, although the response varied among GCMs and RCP scenarios. Pirmoradian et al. (2020) reported WPET values of 0.82 and 0.76 kg m^-^³ for continuously flooded rice under humid and semiarid climates, respectively, and showed that water productivity varied with irrigation regime and climatic condition. Zheng et al. (2020) evaluated rice water footprint together with WPI, WPU, and WPET under future RCP scenarios and reported increasing rice WF trends at two representative sites in China, while most water productivity indicators were projected to decrease, except for some WPU and WPET responses in Kunshan under RCP 2.6 and RCP 4.5. Overall, future rice water productivity should be assessed using both irrigation-based and evapotranspiration-based indicators.

### 3.6 Statistical comparison of water footprint and productivity indicators

The statistical comparison indicated significant projection-period differences for rainfed WFG and rainfed WPET under both RCP scenarios (p = 0.049). For irrigated WFG+B and irrigated WPET, the period effect was close to the significance level under both RCP scenarios (p = 0.096–0.097), whereas IWP did not show a significant period effect (p = 0.716). The paired Wilcoxon signed-rank test showed that the differences between RCP 4.5 and RCP 8.5 were not statistically significant within the projection periods for the evaluated indicators, with p-values ranging from 0.500 to 1.000. The results of the statistical tests are presented in **Table 3**.

**Table 3:** Non-parametric statistical comparison of water footprint and productivity indicators.

Indicator	Conditions	Friedman test p-value		Wilcoxon signed-rank test p-value		
		RCP 4.5	RCP 8.5	2016-2040	2041-2070	2071-2098
WFG+B	Irrigated	0.096	0.096	0.500	1.000	0.750
WFG	Rainfed	0.049*	0.049*	0.500	0.500	0.500
IWP	Irrigated	0.716	0.716	0.750	1.000	0.750
WPET	Irrigated	0.096	0.096	0.500	1.000	1.000
	Rainfed	0.049*	0.049*	0.750	0.500	1.000

WFG+B, green + blue water footprint; WFG, green water footprint; IWP, irrigation water productivity; WPET, evapotranspiration-based water productivity. *p< 0.05.

## 4 Discussion

The study showed that water footprint is a per-unit-production indicator; therefore, the same level of evapotranspiration may result in different footprint values depending on the simulated yield response. This explains why some scenario combinations with relatively high ETa produced lower WFG+B values, whereas rainfed simulations with moderate ETa produced high WFG values when yield remained low. This interpretation is consistent with the crop water footprint perspective, in which water consumption and crop production are evaluated together rather than as independent variables (Mialyk et al., 2024).

The lower irrigated WFG+B values observed after the near-future period should therefore be evaluated as a combined yield–water response, rather than as a direct indication of reduced water demand. Similar complexity has been reported in rice-based climate impact studies. Bocchiola (2015) showed that climate change may alter rice yield and water footprint in the Po Valley through simultaneous changes in temperature, precipitation, and crop water use. Shrestha et al. (2017) also demonstrated that DSSAT-CERES-Rice outputs can be used to connect climate-driven yield response with water footprint assessment. These studies support the interpretation that model-based footprint projections should be assessed through the interaction between crop growth and water balance components.

The high irrigated yields simulated in some late-future scenarios should also be interpreted within the assumptions of the DSSAT-CERES-Rice modelling framework. These values were obtained under full-irrigation conditions and scenario-specific atmospheric CO_2_ concentrations, where water stress was largely minimized. Therefore, the late-future yield increase may reflect the combined influence of CO_2_ enrichment, GCM-specific temperature and radiation patterns, and crop-growth responses represented by the calibrated cultivar parameters. Warmer conditions may also affect phenological development through changes in thermal time accumulation. However, the simulated high yields should be interpreted as conditional model outputs produced under specified climate, CO_2_, irrigation, and cultivar assumptions, rather than as directly attainable field yield targets for semi-arid Mediterranean rice production. Therefore, possible yield overestimation under long-term full-irrigation and elevated CO_2_ conditions should be considered as a source of model uncertainty.

Under irrigated conditions, the green water component still represented a substantial share of WFG+B, despite the application of irrigation water. This finding is agronomically meaningful because irrigated rice production is often discussed mainly through irrigation water requirement, whereas consumptive footprint analysis separates precipitation-derived and irrigation-derived water use. In the Nanliujiang Catchment of China, Yang et al. (2018) found different climate-change responses for rice yield and water footprint components, suggesting that green and blue components should not be treated as fixed proportions. At basin scale, Lin and Chiang (2025) also identified crop- and basin-specific differences in climate-driven changes in crop yield and water footprint. Together, these studies support the present interpretation that green–blue contribution is region- and scenario-dependent. When green and blue consumptive water use is separated using the difference between irrigated and rainfed evapotranspiration, the two simulations may not always represent the same crop development pathway. Under water-stressed rainfed conditions, shorter growth duration, limited canopy development, changes in root water uptake, or earlier senescence may alter the seasonal evapotranspiration pattern. Therefore, the calculated difference between irrigated and rainfed evapotranspiration may partly reflect stress-related crop development responses in addition to irrigation-derived water use. This may introduce some uncertainty into blue water footprint estimation based on paired irrigated–rainfed simulations. Therefore, this approach may tend to overestimate the true blue water footprint component, because the ETa difference between irrigated and rainfed simulations may partly reflect stress-induced reductions in canopy development and growth duration, rather than irrigation-derived water consumption alone. Accordingly, the paired ETa-difference approach may introduce an upward bias in WFB, because part of the irrigated–rainfed ETa gap may arise from stress-related crop development effects under rainfed conditions, including reduced canopy development, earlier senescence, and shortened growth duration, rather than from irrigation-derived consumptive water use alone.

Rainfed conditions produced the highest WFG values in the near-future period, mainly because low simulated yields increased water footprint per unit of production. This result indicates that rainfed rice water footprint is highly sensitive to yield response. The very high rainfed WFG values calculated for 2016–2040 were mainly related to low simulated yields under non-irrigated conditions. In Mediterranean rice production, limited effective rainfall during the growing season and high atmospheric water demand can intensify soil-water stress. These conditions may restrict canopy development, shorten the effective growth period, and reduce biomass accumulation and grain productivity. As a result, the near-future rainfed WFG values increased mainly because simulated yield was reduced, while seasonal evapotranspiration did not decrease at a similar level. In climate–crop assessments, yield reduction is often one of the main reasons why water footprint per unit production increases, even when total seasonal water consumption does not rise proportionally. Arunrat et al. (2020) emphasized that rice water footprint responses under climate change can differ according to farming practice and adaptation capacity. This supports the need to evaluate rainfed and irrigated production separately rather than treating rice water footprint as a single production-wide indicator. Therefore, the rainfed scenario should not be interpreted as a recommended production practice for semi-arid Mediterranean rice cultivation; rather, it represents an extreme non-irrigated baseline used to highlight the role of irrigation in sustaining rice production under water-limited growing-season conditions.

The yield–evapotranspiration relationship observed in this study also demonstrates why footprint and productivity indicators should be interpreted together. WFG+B and WFG describe water consumption per unit production, whereas IWP and WPET describe production per unit water input or water consumption. These indicators therefore provide complementary information. Chukalla et al. (2015) showed that irrigation techniques, irrigation strategies, and mulching can affect evapotranspiration, yield, and consumptive water footprint in irrigated crop production. This is relevant to the present study because future rice water performance depends not only on the amount of water consumed, but also on how effectively that water is converted into yield.

The wide range of IWP values in the present simulations indicates that irrigation-based productivity can be strongly affected by both irrigation amount and yield response. However, IWP alone may be misleading when evaluated without ETa, because it is calculated only from applied irrigation water. WPET provides an additional productivity perspective by relating yield to actual evapotranspiration, including both precipitation-derived and irrigation-derived water consumption. This distinction is particularly important under climate change because precipitation, irrigation requirement, and evapotranspiration may change in different directions across GCMs and projection periods.

The use of a validated DSSAT-CERES-Rice framework strengthened the methodological basis of this assessment. Rather than evaluating future yield alone, the present study translated model-derived yield and evapotranspiration outputs into WFG, WFB, WFG+B, IWP, and WPET. This model-to-indicator transition is consistent with recent efforts to use process-based crop models for water footprint assessment. Mialyk et al. (2024), for example, used a global crop model to estimate crop water use and water footprints for many crops over a long historical period. Although their spatial scale and modelling framework differ from the present study, their approach supports the methodological direction of linking simulated crop growth and water balance outputs with footprint indicators.

From a management perspective, future rice water footprint reduction should not be framed only as a reduction in water input. Reducing water input without maintaining yield may increase water footprint per unit production. Therefore, adaptation strategies should aim to preserve yield while improving the efficiency of water use. Hsieh et al. (2025) recently used DSSAT to evaluate rice yield variation under water-saving scenarios, showing that model-based irrigation scenario analysis can help identify strategies that reduce water use while limiting yield loss. This supports the need to expand the present framework beyond full-irrigation reference and rainfed conditions.

The present study also highlighted the importance of climate-model uncertainty. Differences among HadGEM2-ES, GFDL-ESM2M, and MPI-ESM-MR affected the simulated yield, ETa, irrigation requirement, and water footprint indicators. This suggests that future water footprint assessments should avoid relying on a single climate model where possible. Multi-GCM evaluation provides a more informative range of possible responses and helps distinguish general tendencies from model-specific outcomes.

These findings should be evaluated within the methodological scope of a consumptive water footprint assessment based on validated crop-model outputs. The focus on green and blue components provided a direct basis for evaluating future crop water consumption and water productivity. However, the grey water footprint was not quantified in the present study because its calculation requires pollutant-specific data, including fertilizer and agrochemical loads, leaching or runoff fractions, maximum allowable concentration limits, background concentrations, and receiving-water quality conditions. These variables were not included in the DSSAT-based dataset used in this study, which was developed for yield, evapotranspiration, irrigation requirement, and water-balance assessment. Therefore, the present results represent the consumptive water-use response of Mediterranean rice under future climate scenarios, rather than a full assessment of both water quantity and water quality impacts. Recent water footprint studies have increasingly combined process-based crop or hydrological modelling with spatially and temporally explicit water balance assessment, indicating that future applications can benefit from linking crop-level footprint indicators with basin-scale water availability and water quality constraints (Mialyk et al., 2024; Lin and Chiang, 2025).

The assumption of unchanged soil conditions may also affect the interpretation of long-term blue water-related results. If soil degradation or salinity accumulation develops over time, additional water may be required for leaching, drainage management, or maintaining suitable root-zone conditions. Since these processes were not dynamically represented in the present DSSAT-based framework, the reported WFB values should be interpreted as consumptive blue water estimates under stable soil conditions. Therefore, they may represent an optimistic assessment of long-term blue water pressure, particularly where future salinity-related water requirements could increase.

A further limitation of the present assessment is related to the use of a single cultivar-specific genetic coefficient set for long-term future simulations. The DSSAT-CERES-Rice framework used in this study was calibrated and validated with field data from the 2019–2020 growing seasons, and the same cultivar parameter set was applied across future climate scenarios up to 2098. This approach allowed climate- and water-regime effects to be evaluated under a consistent cultivar assumption. However, it did not account for possible future genetic adaptation, breeding progress for drought or heat tolerance, or the adoption of shorter-duration cultivars. Therefore, the projected yield, water footprint, and water productivity responses should be evaluated within this static cultivar framework.

From an adaptation perspective, the comparison between full-irrigation and rainfed conditions provides a useful framework for evaluating the range of future rice water performance under contrasting water regimes. However, practical management-oriented assessments should also test intermediate irrigation strategies, cultivar responses, planting date adjustments, and threshold-based irrigation scheduling. Although the present assessment compared full-irrigation and rainfed conditions, intermediate water-saving strategies such as AWD and deficit irrigation are also important for adaptation-oriented rice water management. Under AWD, the blue water footprint is generally expected to decrease because less irrigation water is applied. However, the change in total green + blue water footprint depends on the yield response under water-saving irrigation. When yield loss remains limited, AWD can decrease the blue component and improve water productivity. In cases where water-saving irrigation negatively affects crop growth and grain yield, the water footprint per unit production may remain high. Therefore, AWD, deficit irrigation, and irrigation scheduling based on soil-water or plant-water stress limits need to be evaluated in future DSSAT-based studies under Mediterranean rice-growing conditions. Although the present framework compared full-irrigation and rainfed conditions, defining a fixed operational threshold for AWD or deficit irrigation would require additional DSSAT simulations and, preferably, calibration and validation under the corresponding water-saving irrigation practices. Future studies should therefore quantify such thresholds by jointly evaluating irrigation-water reduction, yield response, WFG+B, IWP, and WPET across AWD or deficit-irrigation scenarios. In irrigated crop production, changes in irrigation technique, irrigation strategy, and mulching have been shown to affect evapotranspiration, yield, and consumptive water footprint (Chukalla et al., 2015). For rice, recent DSSAT-based work has also demonstrated that water-saving irrigation scenarios can be evaluated by modifying irrigation intervals and depths while monitoring yield response (Hsieh et al., 2025). Therefore, future research should extend the present framework from footprint diagnosis toward adaptation-oriented scenario testing.

## 5 Conclusions

Future rice water footprint responses were shaped by the interaction between crop water use, evapotranspiration, yield response, green and blue water contributions, and climate-model-specific conditions. Under irrigated conditions, the projected green and blue water footprint components depended on the combined responses of ET, simulated yield, green and blue water contributions, emission pathway, and GCM-specific climate signals. Green + blue water footprint values tended to be highest during the near-future projection period and remained lower in the mid- and late-future periods. However, these lower footprint values should not be interpreted simply as reductions in total crop water use, because water footprint is strongly affected by the joint response of simulated yield and evapotranspiration. The separate analysis of green and blue components also showed that green water remained a substantial contributor to the consumptive water footprint of irrigated rice. Thus, although irrigation water is an important contributor to the water footprint of irrigated rice, it is not the only source of consumptive water use.

Under rainfed conditions, the maximum green water footprint generally occurred during the near-future projection period. This response was mainly related to low simulated yields, which increased the amount of water consumed per unit of rice produced. Therefore, the comparison between irrigated and rainfed conditions indicates that maintaining or improving rice yield levels is a key factor for reducing future rice water footprint, particularly where irrigation water availability is limited or absent.

These findings demonstrate that validated crop model outputs can be used not only to quantify future changes in rice yield, but also to estimate water footprint and water productivity indicators. The analytical framework developed in this study provides a useful basis for assessing future rice water management options in Mediterranean rice-growing environments under climate change.

For Mediterranean rice production, the green and blue water footprint results provide a practical basis for distinguishing between yield losses associated with insufficient rainfall and increasing dependence on irrigation water. High WFG values under rainfed conditions indicate that rainfall alone may not provide a reliable production pathway when yield decreases markedly. In contrast, higher blue water contributions under irrigated conditions show where pressure on irrigation resources may become more critical. Therefore, regional water management plans should combine precise irrigation scheduling, water-saving irrigation practices such as AWD and deficit irrigation, and cultivar selection or breeding efforts targeting drought and heat tolerance. Such an integrated approach may help planners allocate irrigation water more effectively, avoid unnecessary blue water use, and sustain rice production under future Mediterranean climate conditions.

Future studies should extend this framework by evaluating intermediate water-saving irrigation regimes, cultivar adaptation scenarios, soil-related constraints, and water quality-related footprint components to improve the practical applicability of long-term rice water management projections.

## Data Availability

The raw data supporting the conclusions of this article will be made available by the authors, without undue reservation.
